# A Skeletal Muscle Protein That Regulates Endurance

**DOI:** 10.1371/journal.pbio.0020315

**Published:** 2004-08-24

It's a common runner's complaint. Just when you've built up enough strength and endurance to make running fun, those niggling aches and pains won't go away. Every time your foot hits the ground, a force equal to about twice your weight shoots through your body, eventually chipping away at bones, cartilage, muscles, tendons, ligaments, and joints. For those lucky souls who can take the pounding, the main limitation to running performance stems from muscle fatigue. Now, Randall Johnson and colleagues report that a protein found in skeletal muscle profoundly influences muscle endurance.

Running, like any sustained skeletal muscle activity, consumes large quantities of adenosine triphosphate (ATP), a molecule that fuels many essential cell processes. A number of metabolic pathways supply muscle tissue with the ATP needed to power muscle contraction and sustain ongoing exercise. Which pathway predominates depends on factors like speed, duration, and type of activity, as well as on the availability of oxygen, which fluctuates during activity. (For more on muscle cell type and endurance, see the synopsis titled “Gene Targeting Turns Mice Into Long-Distance Runners.”)

Say you start a half-hour run with a sprint. Within a few seconds, your body uses up the oxygen in its muscles and has to switch to anaerobic pathways, which metabolize sugars and fats to regenerate ATP. Aerobic pathways operate inside mitochondria, the cell's major power generators. Anaerobic pathways like glycolysis function in the cytoplasm.

Hypoxia (the physiological state that occurs when oxygen levels drop below normal levels) governs how ATP is recycled and which energy-producing substrates (for example, glucose or fatty acids) are used; it also generates metabolic by-products, like lactic acid, during strenuous exercise. (Runners know the “lactic acid burn” associated with reduced blood pH.) Glycolysis—the primary source of anaerobic energy in animals—uses glucose, stored as glycogen in muscle cells, to produce ATP. When blood oxygen levels drop, the gene transcription factor hypoxia-inducible factor 1α (HIF-1α) triggers the glycolytic pathway.

To understand how HIF-1α regulates skeletal muscle function, Johnson's team generated mice that couldn't express HIF-1α in skeletal muscle. Normal and mutant mice went through exercise routines that included swimming and running on treadmills. After exercise, the normal mice had increased levels of gene transcripts and enzymes involved in glucose transport and metabolism. In the mutant mice, expression of these glycolysis-associated genes and enzymes was significantly lower. The mutants' ATP levels, however, were normal. Without the molecular machinery to engage anaerobic metabolism, their muscles switched to aerobic pathways. The presence of enzymes that respond to reduced ATP levels by increasing mitochondrial ATP production, combined with low levels of lactic acid, confirmed the switch.

During endurance tests, the mutants could swim and run uphill (on treadmills tilted upward) longer than the normal mice, but when it came to running downhill, the normal mice prevailed. Downhill running, it turns out, favors glycolytic metabolism; uphill running and swimming favor oxidative pathways, which the mutants were predisposed toward. But their inappropriate use of this pathway came at a cost. By the final day of a four-day exercise routine, the mutants' run time was significantly shorter and their muscles were clearly damaged.

The mutants displayed a number of the trademark muscle defects seen in human patients with glycolytic processing disorders. These patients often have reduced lactate levels and elevated levels of mitochondrial enzymes, which apparently can cause a second wind and enhance endurance. This inappropriate use of oxidative pathways—which compensates for the inability to trigger glycolysis—may account for the exercise-induced muscle damage associated with these diseases.

These results demonstrate that losing the molecular wherewithal to engage hypoxia response pathways has serious consequences for muscle function during exercise; it can give increased endurance, but at a high price. The mouse model presented here will help researchers explore how muscles normally function in response to low oxygen and how metabolic deficiencies cause debilitating muscle disease.

